# Evaluation of Arterial Spin Labeling MRI—Comparison with ^15^O-Water PET on an Integrated PET/MR Scanner

**DOI:** 10.3390/diagnostics11050821

**Published:** 2021-05-01

**Authors:** Markus Fahlström, Lieuwe Appel, Eva Kumlien, Torsten Danfors, Mathias Engström, Johan Wikström, Gunnar Antoni, Elna-Marie Larsson, Mark Lubberink

**Affiliations:** 1 Department of Surgical Sciences, Uppsala University, 75185 Uppsala, Sweden; lieuwe.appel@akademiska.se (L.A.); torsten.danfors@radiol.uu.se (T.D.); johan.wikstrom@radiol.uu.se (J.W.); elna-marie.larsson@radiol.uu.se (E.-M.L.); mark.lubberink@radiol.uu.se (M.L.); 2 Department of Neuroscience, Uppsala University, 75185 Uppsala, Sweden; eva.kumlien@neuro.uu.se; 3 Applied Science Laboratory, GE Healthcare, 75185 Uppsala, Sweden; mathias.engstrom@ge.com; 4 Department of Medicinal Chemistry, Uppsala University, 75185 Uppsala, Sweden; gunnar.antoni@akademiska.se

**Keywords:** ^15^O-water PET, ASL, CBF, PET/MR, validation

## Abstract

Cerebral blood flow (CBF) measurements are of high clinical value and can be acquired non-invasively with no radiation exposure using pseudo-continuous arterial spin labeling (ASL). The aim of this study was to evaluate accordance in resting state CBF between ASL (CBF_ASL_) and ^15^O-water positron emission tomography (PET) (CBF_PET_) acquired simultaneously on an integrated 3T PET/MR system. The data comprised ASL and dynamic ^15^O-water PET data with arterial blood sampling of eighteen subjects (eight patients with focal epilepsy and ten healthy controls, age 21 to 61 years). ^15^O-water PET parametric CBF images were generated using a basis function implementation of the single tissue compartment model. Cortical and subcortical regions were automatically segmented using Freesurfer. Average CBF_ASL_ and CBF_PET_ in grey matter were 60 ± 20 and 75 ± 22 mL/100 g/min respectively, with a relatively high correlation (*r* = 0.78, *p* < 0.001). Bland-Altman analysis revealed poor agreement (bias = −15 mL/100 g/min, lower and upper limits of agreements = −16 and 45 mL/100 g/min, respectively) with a negative relationship. Accounting for the negative relationship, the width of the limits of agreement could be narrowed from 61 mL/100 g/min to 35 mL/100 g/min using regression-based limits of agreements. Although a high correlation between CBF_ASL_ and CBF_PET_ was found, the agreement in absolute CBF values was not sufficient for ASL to be used interchangeably with ^15^O-water PET.

## 1. Introduction

Cerebral blood flow (CBF) measurements are of high clinical value for various brain disorders such as cerebrovascular disorders, brain tumors, and neurodegenerative diseases [1,2,3]. Current reference standard for CBF measurements is positron emission tomography (PET) with ^15^O-water [3]. Although ^15^O-water PET has proven its usefulness in physiological experiments and clinical assessments [4], it is often considered costly and implementations are limited due to the requirement for an on-site cyclotron and an arterial line for blood sampling. Arterial spin labeling (ASL) is a non-invasive magnetic resonance imaging (MRI)-based CBF measurement technique using the patient’s own water molecules in blood as a freely diffusible tracer. ASL is not a novel technique; the basic principle was already introduced in the early 1990s [5,6,7]. A consensus statement was published in 2015 [8] recommending pseudo-continuous ASL as a labeling strategy with 3D segmented read-out applying background suppression [8].

There are a number of methodological considerations when comparing ^15^O-water PET and ASL acquired CBF. First, comparison studies should be preferably based on simultaneously acquired ^15^O-water PET and ASL CBF measurements using an integrated PET/MR system since the physiological cerebral perfusion state is time dependent [1,9,10,11]. Second, the lack of CT data for attenuation correction of PET images on an integrated PET/MR system needs another approach. Although MRI-based attenuation correction methods are still under investigation, zero-echo time (ZTE)-based attenuation correction has recently been demonstrated to be an adequate method for brain PET/MR applications [12,13,14]. Third, ^15^O-water PET is considered the gold standard for measuring CBF when using a kinetic modeling approach with an arterial input function (AIF) based on arterial sampling [1,15,16]. Although several studies compared CBF measurements with ^15^O-water PET and ASL, none of them fulfilled all three requirements. From those studies conducted on a PET/MR system, the MRI-based attenuation correction methods employed have generally demonstrated inadequate performance compared to ZTE-based attenuation correction. Further, arguing its invasiveness and discomfort, arterial blood sampling was often omitted [9,17]. Moreover, there were obvious differences in study participants and the ASL method used.

The aim of this study was to evaluate accordance in resting state CBF values based on simultaneously acquired ASL (CBF_ASL_) and ^15^O-water PET (CBF_PET_) on an integrated PET/MR system and using ZTE-based attenuation correction and arterial sampling.

## 2. Materials and Methods

### 2.1. Scope and Subjects

In this methodological study, conducted between December 2015 and May 2018, a comparative analysis was performed on, in total, eighteen participants—eight patients with focal epilepsy (5 females, 3 males) with a mean (standard deviation, SD) age of 39 (13) and ten healthy controls (5 females, 5 males) with a mean (SD) age of 40 (12). The patients were given lamotrigine (4 patients) or carbamazepine (6 patients). Two patients were given carbamazepine in combination with clonazepam or valproic acid. No patient had a history of taking levetiracetam. Furthermore, none of the enrolled participants had any intellectual disability. The healthy controls were matched on age and sex with the patients. Recruitment was done by advertisement and no participant had any relations to the hospital or the faculty staff. Each participant was its own control, and thus potential regional differences in CBF due to factors like age, gender and groups of participants were outside the scope of the study. Epilepsy patients were scanned in interictal state. 

The study was done in accordance with the declaration of Helsinki. Approvals were obtained by the Regional Board of Medical Ethics in Uppsala (DNR 2015/187) and the Radiation Ethics Committee at Uppsala University Hospital. After a complete description of the study, and prior to inclusion, all participants signed an informed consent form. 

### 2.2. Data Acquisition

All examinations were performed on an integrated PET/MR (SIGNA, GE Healthcare, Waukesha, WI, USA) which combines a 3T MRI with a time-of-flight capable silicone photomultiplier-based PET scanner [18]. All subjects were scanned in supine position using an eight-channel head coil (MR Instruments Inc., Minneapolis, MN, USA). A 10-min dynamic PET scan was started after automatic bolus injection (1 mL/s during 5 s) of 5 MBq/kg ^15^O-water (max 500 MBq), followed by flushing with 35 mL saline at 2 mL/s. Continuous blood sampling (3 mL/min) was conducted from a radial artery, generally in the non-dominant arm, and blood radioactivity was measured using a Twilite Two blood detector (Swisstrace, Zurich). The blood detector was positioned on the scanner bed as close as possible to the subjects’ wrists to minimise dispersion. During PET scanning, a 3D pseudo-continuous ASL with background suppressed fast spin echo spiral read-out using a PLD of 2025 ms and label duration of 1800 ms was acquired. In addition, the protocol included a high-resolution 3D-T1-weighted (T1w) image and a 3D-T2-weighted fluid attenuated inversion recovery (T2w-FLAIR) as anatomical references and a ZTE image for attenuation correction of PET data. The full set of acquisition parameters for MRI scans is presented in Supplementary Table S1.

### 2.3. Image Reconstruction and Generation of Parametric CBF Images

The ^15^O-water PET images were reconstructed using time-of-flight ordered subset expectation maximization (4 iterations, 28 subsets), with ZTE-based attenuation correction [13,14] and a 5 mm Gaussian post-filter into 22 frames of increasing durations (1 × 10 s, 8 × 5 s, 4 × 10 s, 2 × 15 s, 3 × 20 s, 2 × 30 s, 2 × 60 s) into a 128 × 128 × 89 matrix with 2.34 × 2.34 × 2.81 mm^3^ voxels. The last four minutes of the acquisition were not included due to limited information and increasingly noisy blood data. All subject-specific AIFs were corrected for delay and dispersion [19] and ^15^O-water PET derived parametric CBF images (CBF_PET_) were produced using a basis function implementation of the standard single-tissue compartment model including a fitted blood volume parameter [20]. ASL derived parametric CBF images (CBF_ASL_) were generated according the single compartment model defined by Buxton et al., [21] and recommended by Alsop et al., [8] including a correction term for full proton density reference [22]. 

### 2.4. Post-Processing

T2w-FLAIR, CBF_ASL_ and CBF_PET_-images were co-registered to each subject’s corresponding T1w images. Grey matter (GM) tissue probability maps were segmented based on T1w images and co-registered T2w-FLAIR images. GM maps were defined with a tissue probability fraction above 75%. White matter (WM) maps were disregarded because of the general limitations of ASL in WM [1,8]. All processing steps, as described above, were performed using the SPM12 toolbox (Wellcome Trust Centre for Neuroimaging, London, UK). 

Various volumes of interest (VOI) across the brain were used. The following VOIs were included: cortical (frontal, parietal, occipital, and temporal lobe) and subcortical (caudate, putamen, pallidum, thalamus, amygdala, and hippocampus). VOIs were segmented on 3D-T1w and co-registered T2-FLAIR images using the Freesurfer processing pipeline (version 6.0, http://surfer.nmr.mgh.harvard.edu, accessed on 17 April 2020) [23]. The outline of the VOIs is illustrated in Supplementary Figure S1.

### 2.5. Comparative Analysis

A descriptive analysis was made to compare CBF_ASL_ and CBF_PET_ for both all selected brain regions as well as for clusters of cortical and subcortical regions and whole-brain GM. Agreement between quantitative CBF_ASL_ and CBF_PET_ values was first studied using correlation analysis including Pearson’s product moment correlation coefficient and orthogonal regression. The correlation and regression measures are reported with corresponding 95% confidence interval (CI). Thereafter, Bland-Altman analyses were performed to examine the relationship between the average of CBF_ASL_ and CBF_PET_ and the difference between CBF_ASL_ and CBF_PET_. In addition, bias, expressed as average difference between both methods, was estimated with 95% lower and upper limits of agreement (LoA_L_ and LoA_U_, respectively). Potential relationships between the difference and the average of CBF_PET_ and CBF_ASL_ were identified using linear regression [24], and regression-based LoAs (RLoA_L_ and RLOA_U_) were calculated to account for any potential relationships found [25]. A calculation example for GM is provided in Supplementary Document S1. All statistical tests are two-sided using GraphPad Prism 8 (GraphPad Software, La Jolla, CA, USA).

## 3. Results

### 3.1. Descriptive Analysis

**Figure 1:**
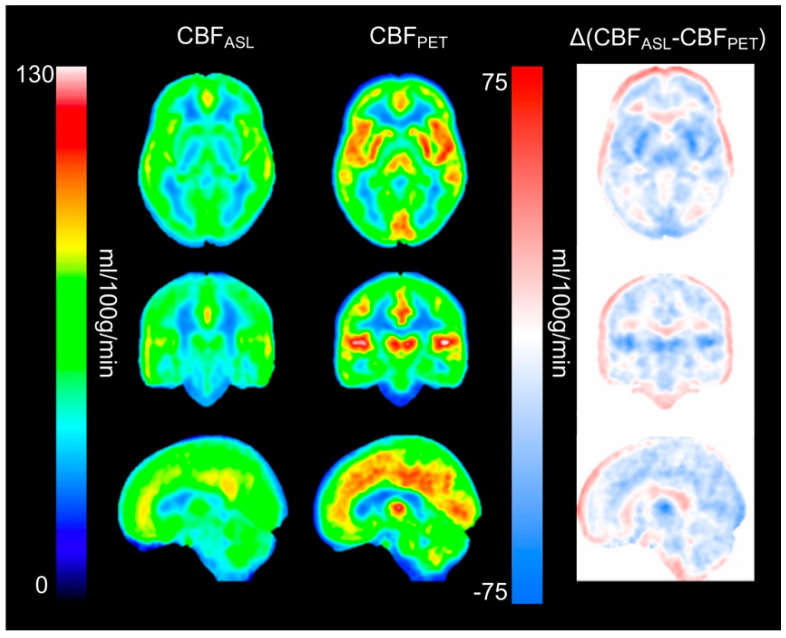
Average parametric CBF_ASL_ and CBF_PET_ images, and differences (CBF_ASL_–CBF_PET_) in MNI template space. Normalization performed with SPM12.

Average parametric CBF_ASL_ and CBF_PET_ images and the differences between both methods are shown in Figure 1. This figure illustrates that CBF_ASL_ resulted in lower values than CBF_PET_, which was consistent throughout the whole GM. Quantitative CBF values are presented for all regions and both methods in Table 1. In GM, average CBF was 75 ± 22 and 60 ± 10 mL/100 g/min for CBF_PET_ and CBF_ASL_, respectively. A larger average difference between CBF_ASL_ and CBF_PET_ was found in subcortical regions compared to cortical regions, especially in caudate, putamen, pallidum, and thalamus. Further, it can be noticed that the variability across subjects, given as SD, was substantially greater for CBF_PET_ compared to CBF_ASL_.

### 3.2. Correlation and Regression

**Figure 2:**
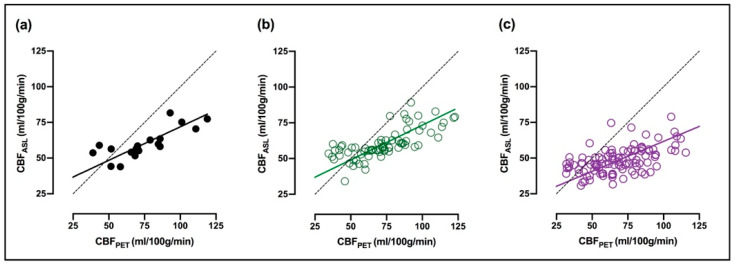
Relation between CBF_ASL_ and CBF_PET_ and corresponding orthogonal regression (solid line) in (**a**) GM, (**b**) cortical regions (green, open circles), and (**c**) subcortical regions (purple, open circles). The dashed line is the line of identity.

Correlations between CBF_ASL_ and CBF_PET_ are shown for GM as well as clusters of cortical- and subcortical regions in Figure 2. Correlations between CBF_ASL_ and CBF_PET_ are given for all regions in Table 1. Similar and positive correlations between CBF_ASL_ and CBF_PET_ were found for GM (Figure 2a) and the cluster of cortical regions (Figure 2b), 0.78 and 0.73, respectively. An obvious lower correlation between both methods was found for subcortical regions (Figure 2c r = 0.53). Correlations in subcortical regions varied between 0.42 (pallidum) and 0.66 (caudate).

### 3.3. Analysis of Agreement between CBF_ASL_ and CBF_PET_

**Figure 3:**
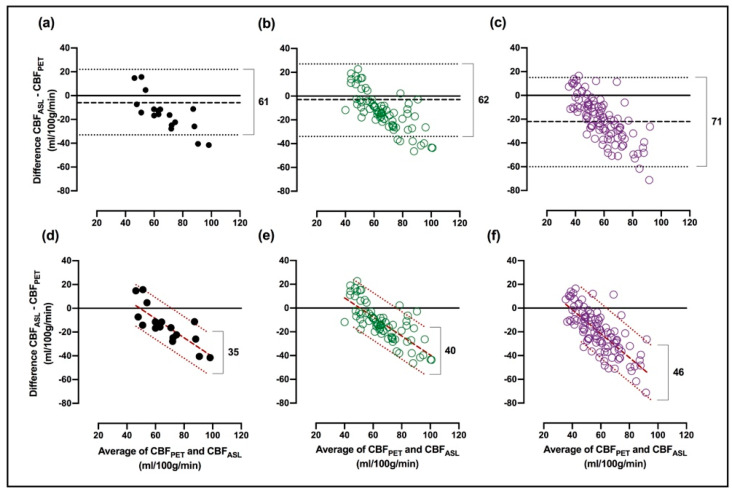
Bland–Altman plot including bias (black, dashed lines) and limits of agreements (black, dotted lines) for (**a**) GM, (**b**) cortical regions (green, closed circles), and (**c**) subcortical regions (purple, open circles). For comparison, regression-based limits of agreement (dark red, dotted lines) and regression (dark red, dashed line) for (**d**) GM, (**e**) cortical- (green, closed circles), and (**f**) subcortical regions (purple, open circles). The width of the regression-based- and ordinary limits of agreement are given mL/100 g/min in each graph.

Bland-Altman plots displayed a negative, nearly linear relationship for GM (Figure 3a) as well as cortical- and subcortical regions (Figure 3b,c, respectively). Bias was −15, −13 and −20 mL/100 g/min for GM, cortical- and subcortical regions, respectively. Quantitative results of the Bland–Altman analysis are given for individual regions in Table 2. The differences between the considered cortical regions were relatively small. In contrast, subcortical regions showed a large variation. The highest bias was found in putamen, pallidum, and thalamus (−34 to −26 mL/100 g/min) and lowest bias in amygdala and hippocampus (−10 to −6 mL/100 g/min).

### 3.4. Regression-Based Limits of Agreements

A negative relationship of the difference on the average of CBF_PET_ and CBF_ASL_ was found and RLoAs were calculated for all regions. The bias remains unchanged using RLoAs. The width between upper and lower LoAs changed from 61 mL/100 g/min to 35 mL/100 g/min in GM using RLoAs. In cortical- and subcortical regions, the width changed from 62 mL/100 g/min to 40 mL/100 g/min and from 71 mL/100 g/min to 46 mL/100 g/min, respectively (compare Figure 3a–c with Figure 3c–f). A consistent narrowing was found for all regions when using RLoAs instead of ordinary LoAs. Quantitative results from the RLoA method are given for individual regions in Table 3.

## 4. Discussion

This study evaluated the agreement between CBF_ASL_ and CBF_PET_ derived from parametric images allowing a quantitative comparison between both methods. We found a relatively high correlation between CBF_ASL_ and CBF_PET_ in GM in comparison to previously published work. However, the agreement between CBF_ASL_ and CBF_PET_ was poor. We observed a negative proportional bias between the difference and average of CBF_PET_ and CBF_ASL_ in all regions. This is also apparent in the orthogonal regression, where the slope is less than 1 for all regions, i.e., the difference will increase as the average of CBF_PET_ and CBF_ASL_ increases. There is also an apparent underestimation of CBF_ASL_ in subcortical regions, which may be caused by shorter than assumed T1 relaxation and earlier arrival of labeled blood compared to cortical regions [9,22]. 

**Figure 4:**
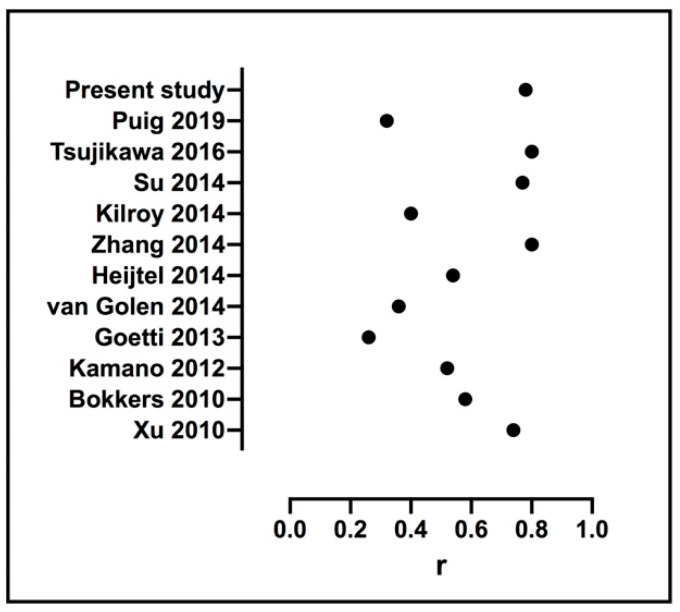
Previously reported correlation coefficients between GM CBF derived from ^15^O-water PET and ASL.

Previous studies comparing ASL and ^15^O-water PET have reported correlation coefficients in GM ranging from 0.26 to 0.81 [4,10,11,26,27,28,29,30,31,32,33] (see Figure 4). In our study, we found correlations between CBF_ASL_ and CBF_PET_ varying between 0.42 in pallidum and 0.83 in frontal cortex. Two of the previous studies performed arterial blood sampling and used an integrated PET/MR. Zhang et al. [11]. reported a correlation of 0.80 in GM and 0.61 to 0.87 in cortical- and subcortical regions comparing simultaneously acquired CBF measurements. Although the reported correlation coefficients agree with ours, Zhang et al. [11]. found generally higher values for CBF_ASL_ than for CBF_PET_, which is contrary to our results. However, Zhang et al. [11], used template-based attenuation correction, which might underestimate PET tracer uptake [14,34]. Puig et al. [4]. compared simultaneously acquired CBF measurements during rest in healthy subjects and found a correlation of 0.32. Correlations in cortical- and subcortical regions were reported using combined data from rest and altered perfusion states, so any comparisons to our study are hard to make. Both Zhang et al. [11] and Puig et al. [4] also performed a Bland–Altman analysis and reported a bias (LoA_L_ and LoA_U_) of 15 (−5 and 25, width 30) and 0 (−15 and 15, width 30) mL/100 g/min in GM [4,11], respectively, compared to −15 (−45 and 16, width 61) mL/100 g/min in the present study. No regression analysis was performed in any of the two studies. Furthermore, Bland–Altman analysis of cortical and subcortical regions was omitted or included several altered perfusion states in the two above mentioned studies, so no further comparison to our results is possible except for whole GM. 

When using RLoAs, the width between the upper and lower LoAs is drastically narrowed, indicating that the negative relationship between the difference and the average of CBF_PET_ and CBF_ASL_ has a high impact on data interpretation. In GM, the width of the LoAs went from 61 mL/100 g/min to 35 mL/100 g/min, LoA_U_ and LoA_L_ were 16 and −45 mL/100 g/min, respectively. RLoAs are uniform around the regression line; therefore the corresponding upper and lower limits were 18 and −18 mL/100 g/min, which is more comparable to the LoAs reported by other investigations. 

Still, given the width of the RLoAs compared to the normal-range CBF values, the agreement between CBF_ASL_ and CBF_PET_ is not sufficient to be used interchangeably for measuring absolute and comparable CBF. However, re-scaling of ASL values using the relation between CBF_PET_ and CBF_ASL_ found in the present work could be considered. As different re-scaling would be required in cortical and subcortical regions, this may not be a feasible way to proceed.

We report an average CBF_PET_ in GM at 75 mL/100 g/min. In contrast, previous studies using ^15^O-water PET with arterial sampling have reported CBF values in GM ranging from 37 to 67 mL/100 g/min in healthy subjects [11,28,35,36,37,38,39,40,41]. Thus, current published normal-range CBF measured by ^15^O-water PET with arterial blood sampling shows large variations [36,42]. A generally accepted and often-cited average normal whole-brain CBF value in younger adults is 50 mL/100 g/min [43]. Moreover, average whole-brain, GM and WM CBF values of 50, 80, and 20 mL/100 g/min in neurologically normal subjects were early established using the Kety-Schmidt method with intra-arterial injection [36,44,45], which is in line with the CBF_PET_ values found in the current work. We acknowledge that our reported average CBF_PET_ in GM are high compared to other investigations, however, during quality control of our data we found no technical explanation. Of note, in subjects/patients with high average CBF_PET_, we also found high CBF_ASL_. Thus, a physiological explanation cannot be ruled out, but appears to be unlikely. 

CBF derived from ASL is inherently dependent on the sequence implantation, vendor, and quantification method used. Therefore, caution is advised when generalizing the results and conclusions found here. Other investigations have stressed the importance of the PLD for the quantification of CBF with ASL. In studies where ASL and ^15^O-water PET were compared in patients with cerebrovascular diseases affecting the blood transit time, PLD appeared to be a critial parameter that can affect the results. However, all subjects and patients in this study are regarded to have a normal blood transit time. Hence we have used a PLD of 2000 ms as described by Alsop et al. [8]. 

We evaluated agreement in the normal CBF range. In addition to ten healthy volunteers, we included eight patients with epilepsy. However, interictal focal hypoperfusion is expected to have a negligible impact on our results since we used mostly large VOIs. Moreover, an unpaired t-test between patients with epilepsy and healthy subjects did not reveal any significant differences for any region (results not shown). A thorough investigation of potential differences was outside the scope of the current study and therefore not reported in detail.

## 5. Conclusions

Although a high correlation between CBF_ASL_ and CBF_PET_ was found, the agreement in absolute and comparable CBF values was not sufficient for ASL to be used interchangeably with ^15^O-water PET. 

## Figures and Tables

**Table 1:** Descriptive statistics of CBF_PET_ and CBF_ASL_ with correlation and slope from the correlation analysis including orthogonal regression.

	Region	CBF_PET_	CBF_ASL_	*r* [95% CI]	Slope [95% CI]	*p*-Value
	GM	75 (22)	60 (10)	0.78 [0.50, 0.92]	0.47 [0.23, 0.71]	<0.01
	Cortical	73 (22)	60 (10)	0.73 [0.60, 0.82]	0.49 [0.35, 0.62]	<0.01
	Subcortical	68 (21)	48 (9)	0.53 [0.38, 0.66]	0.42 [0.27, 0.57]	<0.01
Cortical	Frontal	74 (23)	62 (10)	0.83 [0.58, 0.93]	0.42 [0.25, 0.59]	<0.01
	Occipital	74 (22)	57 (10)	0.68 [0.31, 0.87]	0.48 [0.10, 0.87]	<0.01
	Parietal	76 (23)	61 (11)	0.75 [0.44, 0.90]	0.49 [0.17, 0.81]	<0.01
	Temporal	66 (20)	60 (11)	0.78 [0.48, 0.91]	0.53 [0.28, 0.78]	<0.01
Subcortical	Caudate	62 (20)	46 (8)	0.66 [0.28, 0.86]	0.39 [0.04, 0.74]	<0.01
	Putamen	85 (22)	50 (7)	0.60 [0.19, 0.84]	0.31 [−0.01, 0.63]	<0.01
	Pallidum	67 (18)	40 (6)	0.42 [−0.06, 0.74]	0.26 [−0.14, 0.65]	0.08
	Thalamus	80 (21)	54 (10)	0.62 [0.21, 0.84]	0.48 [0.09, 0.87]	<0.01
	Amygdala	55 (16)	49 (9)	0.63 [0.23, 0.85]	0.61 [−0.10, 1.40]	<0.01
	Hippocampus	60 (14)	50 (8)	0.65 [0.26, 0.85]	0.54 [0.06, 1.02]	<0.01

**Table 2:** Bland–Altman analysis with slope from the linear regression of the difference between CBF_ASL_ and CBF_PET_ on the average of CBF_ASL_ and CBF_PET_.

	Region	Bias [95% CI]	LOA_L_ [95% CI]	LOA_U_ [95% CI]	Slope [95% CI]	*p*-Value
	GM	−15 [–22, –7]	−45 [−59, −32]	16 [2, 29]	−0.80 [−1.12, −0.49]	<0.01
	Cortical	−13 [−16, −9]	−44 [−50, −37]	18 [12, 25]	−0.80 [−0.96, −0.64]	<0.01
	Subcortical	−20 [−23, −16]	−56 [−50, −61]	16 [10, 22]	−1.00 [−1.18, −0.84]	<0.01
Cortical	Frontal	−12 [−20, −4]	−44 [−58, −30]	19 [5, 33]	−0.88 [−1.15, −0.61]	<0.01
	Occipital	−17 [−25, −9]	−50 [−64, −35]	15 [1, 30]	−0.83 [−1.23, −0.44]	<0.01
	Parietal	−15 [−23, −7]	−47 [−61, −33]	17 [3, 31]	−0.77 [−1.12, −0.43]	<0.01
	Temporal	−6 [−13, 1]	−32 [−44, −21]	20 [9, 32]	−0.67 [−1.00, −0.33]	<0.01
Subcortical	Caudate	−16 [−24, −8]	−47 [−60, −33]	14 [1, 28]	−1.01 [−1.38, −0.64]	<0.01
	Putamen	−34 [−44, −25]	−71 [−87, −55]	2 [−14, 18]	−1.22 [−1.58, −0.86]	<0.01
	Pallidum	−27 [−35, −19]	−60 [−74, −45]	6 [−9, 20]	−1.36 [−1.79, −0.93]	<0.01
	Thalamus	−26 [−34, −18]	−59 [−73, −44]	7 [−8, 21]	−0.85 [−1.29, −0.41]	<0.01
	Amygdala	−6 [−12, −1]	−30 [−41, −20]	17 [7, 28]	−0.62 [−1.08, −0.15]	0.01
	Hippocampus	−10 [−15, −4]	−32 [−42, −22]	13 [3, 23]	−0.70 [−1.14, −0.25]	<0.01

**Table 3:** Slope and intercept of the upper and lower regression-based limits of agreement (RLOA_U_ and RLOA_L_, respectively) with slope and intercept from the regression analysis of the difference between CBF_ASL_–CBF_PET_ on average of CBF_ASL_ + CBF_PET_. A calculation example is given in Supplementary Document S1.

	Region	Linear Regression		RLOA_L_		RLOA_U_	
		Slope	Intercept	Slope	Intercept	Slope	Intercept
	GM	−0.80	40	−0.80	22	−0.80	57
	Cortical	−0.80	41	−0.80	21	−0.80	61
	Subcortical	−1.00	39	−1.00	18	−1.00	64
Cortical	Frontal	−0.88	47	−0.88	30	−0.88	64
	Occipital	−0.83	38	−0.83	17	−0.83	58
	Parietal	−0.77	38	−0.77	19	−0.77	57
	Temporal	−0.67	36	−0.67	18	−0.67	54
Subcortical	Caudate	−1.01	39	−1.01	20	−1.01	58
	Putamen	−1.22	48	−1.22	30	−1.22	66
	Pallidum	−1.36	46	−1.36	29	−1.36	63
	Thalamus	−0.85	31	−0.85	10	−0.85	52
	Amygdala	−0.62	26	−0.62	7	−0.62	44
	Hippocampus	−0.70	29	−0.70	12	−0.70	46

## Data Availability

The datasets analyzed during the current study are available from the corresponding author on reasonable request.

## Supplementary Materials

The following are available online at https://www.mdpi.com/article/10.3390/diagnostics11050821/s1, Table S1: Acquisition parameters of MRI scans, Figure S1: Representative example of VOI definition for a healthy subject, Document S1: Calculation of regression-based limits of agreement (RLoA)–example grey matter.

## References

[1] Fan A.P., Jahanian H., Holdsworth S.J., Zaharchuk G. (2016). Comparison of Cerebral Blood Flow Measurement with [^15^O]-Water Positron Emission Tomography and Arterial Spin Labeling Magnetic Resonance Imaging: A Systematic Review. Br. J. Pharmacol..

[2] Haller S., Zaharchuk G., Thomas D.L., Lovblad K.-O., Barkhof F., Golay X. (2016). Arterial Spin Labeling Perfusion of the Brain: Emerging Clinical Applications. Radiology.

[3] Zhang J. (2016). How Far is Arterial Spin Labeling MRI from a Clinical Reality? Insights from Arterial Spin Labeling Comparative Studies in Alzheimer’s Disease and other Neurological Disorders. J. Magn. Reson. Imaging.

[4] Puig O., Henriksen O.M., Vestergaard M.B., Hansen A.E., Andersen F.L., Ladefoged C.N., Rostrup E., Larsson H.B., Lindberg U., Law I. (2019). Comparison of Simultaneous Arterial Spin Labeling MRI and (15)O-H_2_O PET Measurements of Regional Cerebral Blood Flow in Rest and Altered Perfusion States. J. Cereb. Blood Flow Metab..

[5] Detre J.A., Leigh J.S., Williams D.S., Koretsky A.P. (1992). Perfusion Imaging. Magn. Reson. Med..

[6] Roberts D.A., Detre J.A., Bolinger L., Insko E.K., Leigh J.J.S. (1994). Quantitative Magnetic Resonance Imaging of Human Brain Perfusion at 1.5 T using Steady-State Inversion of Arterial Water. Proc. Natl. Acad. Sci. USA.

[7] Williams D.S., Detre J.A., Leigh J.S., Koretsky A.P. (1992). Magnetic Resonance Imaging of Perfusion using Spin Inversion of Arterial Water. Proc. Natl. Acad. Sci. USA.

[8] Alsop D.C., Detre J.A., Golay X., Günther M., Hendrikse J., Hernandez-Garcia L., Lu H., MacIntosh B.J., Parkes L.M., Smits M. (2015). Recommended Implementation of Arterial Spin-Labeled Perfusion MRI for Clinical Applications: A Consensus of the ISMRM Perfusion Study Group and the European Consortium for ASL in Dementia. Magn. Reson. Med..

[9] Fan A.P., Guo J., Khalighi M.M., Gulaka P.K., Shen B., Park J.H., Gandhi H., Holley D., Rutledge O., Singh P. (2017). Long-Delay Arterial Spin Labeling Provides More Accurate Cerebral Blood Flow Measurements in Moyamoya Patients. Stroke.

[10] Heijtel D., Mutsaerts H., Bakker E., Schober P., Stevens M., Petersen E., van Berckel B., Majoie C., Booij J., van Osch M. (2014). Accuracy and Precision of Pseudo-Continuous Arterial Spin Labeling Perfusion during Baseline and Hypercapnia: A Head-to-Head Comparison with ^15^O H_2_O Positron Emission Tomography. NeuroImage.

[11] Zhang K., Herzog H., Mauler J., Filss C., Okell T.W., Kops E.R., Tellmann L., Fischer T., Brocke B., Sturm W. (2014). Comparison of Cerebral Blood Flow Acquired by Simultaneous [^15^O]Water Positron Emission Tomography and Arterial Spin Labeling Magnetic Resonance Imaging. Br. J. Pharmacol..

[12] Sekine T., Ter Voert E.E.G.W., Warnock G., Buck A., Huellner M.W., Veit-Haibach P., Delso G. (2016). Clinical Evaluation of Zero-Echo-Time Attenuation Correction for Brain 18F-FDG PET/MRI: Comparison with Atlas Attenuation Correction. J. Nucl. Med..

[13] Wiesinger F., Bylund M., Yang J., Kaushik S., Shanbhag D., Ahn S., Jonsson J.H., Lundman J.A., Hope T., Nyholm T. (2018). Zero TE-Based Pseudo-CT Image Conversion in the Head and its Application in PET/MR Attenuation Correction and MR-Guided Radiation Therapy Planning. Magn. Reson. Med..

[14] Sousa J.M., Appel L., Engström M., Papadimitriou S., Nyholm D., Larsson E.-M., Ahlström H., Lubberink M. (2018). Evaluation of Zero-Echo-Time Attenuation Correction for Integrated PET/MR Brain Imaging—Comparison to Head Atlas and 68Ge-Transmission-Based Attenuation Correction. EJNMMI Phys..

[15] Koopman T., Yaqub M., Heijtel D.F.R., Nederveen A.J., Van Berckel B.N.M., Lammertsma A.A., Boellaard R. (2017). Semi-Quantitative Cerebral Blood Flow Parameters Derived from Non-Invasive [^15^O]H_2_O PET Studies. Br. J. Pharmacol..

[16] Lammertsma A.A., Cunningham V.J., Deiber M.P., Heather J.D., Bloomfield P.M., Nutt J., Frackowiak R.S.J., Jones T. (1990). Combination of Dynamic and Integral Methods for Generating Reproducible Functional CBF Images. Br. J. Pharmacol..

[17] Okazawa H., Higashino Y., Tsujikawa T., Arishima H., Mori T., Kiyono Y., Kimura H., Kikuta K.-I. (2018). Noninvasive Method for Measurement of Cerebral Blood Flow using O-15 Water PET/MRI with ASL Correlation. Eur. J. Radiol..

[18] Grant A.M., Deller T.W., Khalighi M.M., Maramraju S.H., Delso G., Levin C.S. (2016). NEMA NU 2-2012 Performance Studies for the SiPM-Based ToF-PET Component of the GE SIGNA PET/MR System. Med Phys..

[19] Meyer E. (1989). Simultaneous Correction for Tracer Arrival Delay and Dispersion in CBF Measurements by the H_2_^15^O Autoradio-graphic Method and Dynamic PET. J. Nucl. Med..

[20] Boellaard R., Knaapen P., Rijbroek A., Luurtsema G.J.J., Lammertsma A.A. (2005). Evaluation of Basis Function and Linear Least Squares Methods for Generating Parametric Blood Flow Images Using ^15^O-Water and Positron Emission Tomography. Mol. Imaging Biol..

[21] Buxton R.B., Frank L.R., Wong E.C., Siewert B., Warach S., Edelman R.R. (1998). A General Kinetic Model for Quantitative Per-fusion Imaging with Arterial Spin Labeling. Magn. Reson. Med..

[22] Dai W., Robson P.M., Shankaranarayanan A., Alsop D.C. (2012). Reduced Resolution Transit Delay Prescan for Quantitative Con-tinuous Arterial Spin Labeling Perfusion Imaging. Magn. Reson. Med..

[23] Fischl B., Salat D.H., Busa E., Albert M., Dieterich M., Haselgrove C., van der Kouwe A., Killiany R., Kennedy D., Klaveness S. (2002). Whole Brain Segmentation: Automated Labeling of Neuroanatomical Structures in the Human Brain. Neuron.

[24] Bland J.M., Altman D.G. (1986). Statistical Methods for Assessing Agreement between two Methods of Clinical Measurement. Lancet.

[25] Bland J.M., Altman D.G. (1999). Measuring Agreement in Method Comparison Studies. Stat. Methods Med Res..

[26] Bokkers R.P.H., Bremmer J.P., Van Berckel B.N.M., Lammertsma A.A., Hendrikse J., Pluim J.P.W., Kappelle L.J., Boellaard R., Klijn C.J.M. (2009). Arterial Spin Labeling Perfusion MRI at Multiple Delay Times: A Correlative Study with H2^15^O Positron Emission Tomography in Patients with Symptomatic Carotid Artery Occlusion. Br. J. Pharmacol..

[27] Goetti R., Warnock G., Kuhn F.P., Guggenberger R., O’Gorman R., Buck A., Khan N., Scheer I. (2013). Quantitative Cerebral Perfusion Imaging in Children and Young Adults with Moyamoya Disease: Comparison of Arterial Spin-Labeling-MRI and H_2_[^15^O]-PET. Am. J. Neuroradiol..

[28] Van Golen L.W., Kuijer J.P., Huisman M.C., Ijzerman R.G., Barkhof F., Diamant M., Lammertsma A.A. (2013). Quantification of Cerebral Blood Flow in Healthy Volunteers and Type 1 Diabetic Patients: Comparison of MRI Arterial Spin Labeling and [^15^O]H_2_O Positron Emission Tomography (PET). J. Magn. Reson. Imaging.

[29] Xu G., Rowley H.A., Wu G., Alsop D.C., Shankaranarayanan A., Dowling M., Christian B.T., Oakes T.R., Johnson S.C. (2009). Reliability and Precision of Pseudo-Continuous Arterial Spin Labeling Perfusion MRI on 3.0 T and Comparison with 15 O-Water PET in Elderly Subjects at Risk for Alzheimer’s Disease. NMR Biomed..

[30] Kamano H., Yoshiura T., Hiwatashi A., Abe K., Togao O., Yamashita K., Honda H. (2013). Arterial Spin Labeling in Patients with Chronic Cerebral Artery Steno-Occlusive Disease: Correlation with ^15^O-PET. Acta Radiol..

[31] Kilroy E., Apostolova L., Liu C., Yan L., Ringman J., Wang D.J. (2013). Reliability of Two-Dimensional and Three-Dimensional Pseudo-Continuous Arterial Spin Labeling Perfusion MRI in Elderly Populations: Comparison with 15o-Water Positron Emission Tomography. J. Magn. Reson. Imaging.

[32] Su Y., Vlassenko A., Blazey T., Ances B., Snyder A., Priatna A., Benzinger T., Raichle M. (2014). Comparison of Cerebral Blood Flow Measurement Obtained from Simultaneously Acquired ASL and O15-Water PET. J. Nucl. Med..

[33] Tsujikawa T., Kimura H., Matsuda T., Fujiwara Y., Isozaki M., Kikuta K.-I., Okazawa H. (2016). Arterial Transit Time Mapping Obtained by Pulsed Continuous 3D ASL Imaging with Multiple Post-Label Delay Acquisitions: Comparative Study with PET-CBF in Patients with Chronic Occlusive Cerebrovascular Disease. PLoS ONE.

[34] Sekine T., Buck A., Delso G., Ter Voert E.E.G.W., Huellner M., Veit-Haibach P., Warnock G. (2015). Evaluation of Atlas-Based Attenuation Correction for Integrated PET/MR in Human Brain: Application of a Head Atlas and Comparison to True CT-Based Attenuation Correction. J. Nucl. Med..

[35] Coles J.P., Fryer T.D., Bradley P.G., Nortje J., Smielewski P., Rice K., Clark J.C., Pickard J.D., Menon D.K. (2005). Intersubject Variability and Reproducibility of ^15^O PET Studies. Br. J. Pharmacol..

[36] Henriksen O.M., Larsson H.B., Hansen A.E., Grüner J.M., Law I., Rostrup E. (2012). Estimation of Intersubject Variability of Cerebral Blood Flow Measurements using MRI and Positron Emission Tomography. J. Magn. Reson. Imaging.

[37] Qiu D., Straka M., Zun Z., Bammer R., Moseley M.E., Zaharchuk G. (2012). CBF Measurements using Multidelay Pseudocontinuous and Velocity-Selective Arterial Spin Labeling in Patients with Long Arterial Transit Delays: Comparison with Xenon CT CBF. J. Magn. Reson. Imaging.

[38] Ye F.Q., Berman K.F., Ellmore T., Esposito G., van Horn J.D., Yang Y., Duyn J., Smith A.M., Frank J.A., Weinberger D.R. (2000). H_2_^15^O PET Validation of Steady-State Arterial Spin Tagging Cerebral Blood Flow Measurements in Humans. Magn. Reson. Med..

[39] Grandin C., Bol A., Smith A., Michel C., Cosnard G. (2005). Absolute CBF and CBV Measurements by MRI Bolus Tracking before and after Acetazolamide Challenge: Repeatabilily and Comparison with PET in Humans. NeuroImage.

[40] Hattori N., Huang S.-C., Wu H.-M., Liao W., Glenn T.C., Vespa P.M., Phelps E.M., Hovda A.D., Bergsneider M. (2004). Acute Changes in Regional Cerebral 18F-FDG Kinetics in Patients with Traumatic Brain Injury. J. Nucl. Med..

[41] Rostrup E., Knudsen G.M., Law I., Holm S., Larsson H.B., Paulson O.B. (2005). The Relationship between Cerebral Blood Flow and Volume in Humans. NeuroImage.

[42] Ishii Y., Thamm T., Guo J., Khalighi M.M., Wardak M., Holley D., Gandhi H., Park J.H., Shen B., Steinberg G.K. (2020). Simultaneous Phase-Contrast MRI and PET for Noninvasive Quantification of Cerebral Blood Flow and Reactivity in Healthy Subjects and Patients with Cerebrovascular Disease. J. Magn. Reson. Imaging.

[43] Lassen N.A. (1985). Normal Average Value of Cerebral Blood Flow in Younger Adults is 50 ml/100 g/min. Br. J. Pharmacol..

[44] Hoedt-Rasmussen K. (1967). Regional Cerebral Blood flow. The Intra-Arterial Injection Method. Acta Neurol. Scand..

[45] Kety S.S., Schmidt C.F. (1948). The Nitrous Oxide Method for the Quantitative Determination of Cerebral Blood Flow in Man: Theory, Procedure and Normal Values 1. J. Clin. Investig..

