# Supplementary material for: Evaluation of Arterial Spin Labeling MRI—Comparison with 15O-Water PET on an Integrated PET/MR Scanner
Source: Diagnostics (Basel). 2021 May 1;11(5):821. doi: 10.3390/diagnostics11050821 (PMC8147295; doi:10.3390/diagnostics11050821)
Supplement: Supplementary file 1 [file diagnostics-11-00821-s001.zip › diagnostics-1204943-supplementary.pdf]

Table S1: Acquisition parameter of MRI scans

Name	Sequence type	Orientation	TR/TE/TI/FA	Matrix (mm)	Slices	Other
T1W	3D-GRE	SAG	8.6/3.2/450/12	1x0.488x0.488	178	NA
T2-FLAIR	3D-TSE	SAG	7500/121/2147/90	0.6x0.488x0.488	296	NA
ASL	3D Pseudo-Continuous FSE	TRA	4852/10.7/NA/90	1.875x1.875x4	36	PLD = 2025 LD = 1800 No flow crushing gradients
ZTE	RUFIS	TRA	0.7/0/NA/0.8	2.4x2.4x2.4	110	NA

ASL = Arterial Spin Labeling; NA = Not applicable; TR = Repetition Time (ms); TE = Echo Time (ms); TI = Inversion Time (ms);

FA = excitation Flip Angle (degrees); TRA = Transversal; SAG = Sagittal; PLD = Post Label Delay (ms); LD = Label Duration (ms);

FSE = Fast Spin Echo; RUFIS = Rotating UltraFast Imaging Sequence

Figure S1. Representative example of VOI definition for a healthy subject

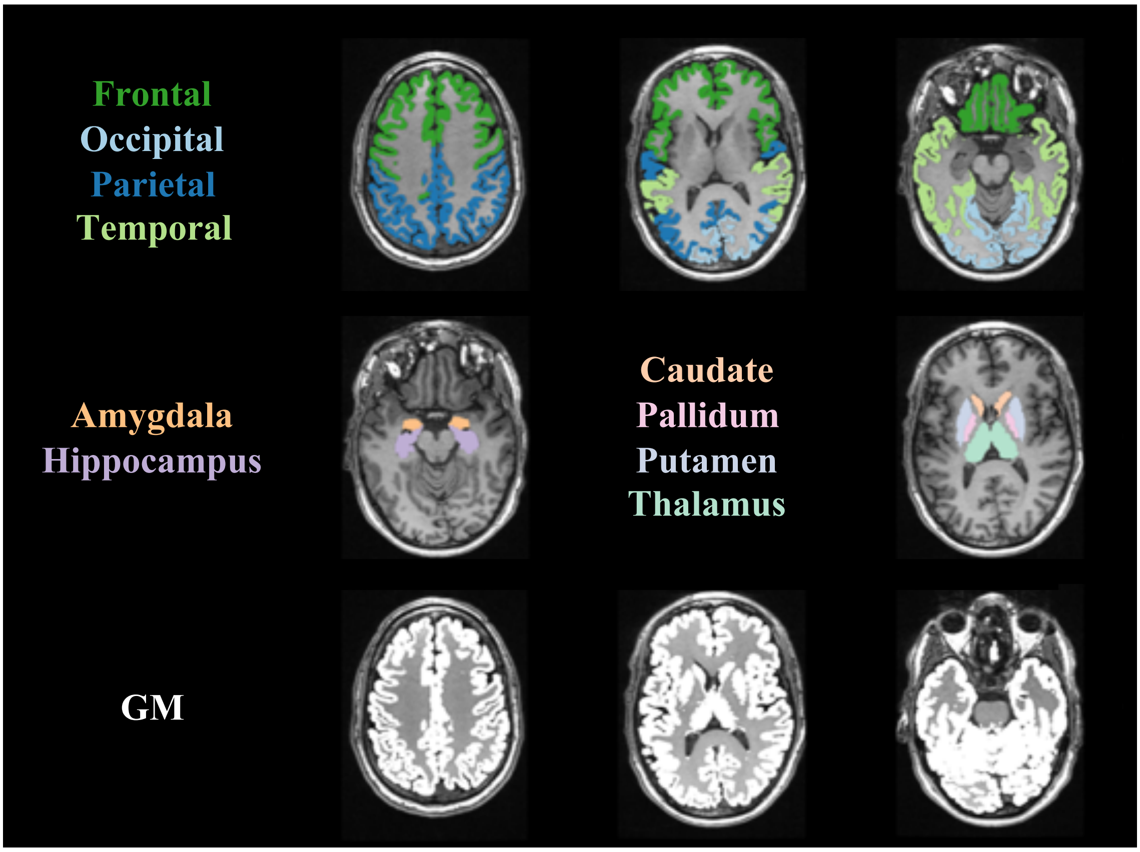

Document S1. Calculation of Regression-based Limits of Agreement (RLoA) – example grey matter

Regression equation of the differences in CBF_{ASL} and CBF_{PET} (D) on the average of CBF_{PET} and CBF_{ASL} (A) was

$$D = -0.80A + 39\ (P < 0.001)$$

The regression equation of the absolute values of the regression residuals (R_{ABS}) on A was

$$R_{ABS} = -0.08A + 13\ (P = 0.3641)$$

If regression of R_{ABS} on A is not significant the standard deviation of R_{ABS} can be estimated as

$$SD_R = \sqrt{\pi/2} \times \bar{R}_{ABS} = \sqrt{\pi/2} \times 7.2 = 9.04$$

Regression-based upper and lower limits of agreement is then

$$D \pm 1.96 \times SD_R$$

Which is equal to

$$RLoA_L = -0.80A + 39 - (1.96 \times 9.04)$$

$$RLoA_U = -0.80A + 39 + (1.96 \times 9.04)$$

These linear equations are plotted on the Bland-Altman plots.
